# Structural Organizations of Qβ and MS2 Phages Affect Capsid Protein Modifications by Oxidants Hypochlorous Acid and Peroxynitrite

**DOI:** 10.3389/fmicb.2020.01157

**Published:** 2020-06-03

**Authors:** Guillaume Bastin, Pauline Loison, Lionel Vernex-Loset, François Dupire, Julie Challant, Didier Majou, Nicolas Boudaud, Gabriel Krier, Christophe Gantzer

**Affiliations:** ^1^Université de Lorraine, CNRS, LCPME, Nancy, France; ^2^ACTALIA, Food Safety Department, Saint-Lô, France; ^3^Université de Lorraine, LCP-A2MC, EA 4632, Metz, France; ^4^Université de Lorraine, L2CM, CNRS, Nancy, France; ^5^ACTIA, 16 rue Claude Bernard, Paris, France

**Keywords:** virus structure, oxidant, Qβ, MS2, inactivation, cross-link, hypochlorous acid, peroxynitrite

## Abstract

Pathogenic enteric viruses and bacteriophages such as Qβ and MS2 are transmitted through the fecal-oral route. However, oxidants such as peroxynitrite (ONOOH) and hypochlorous acid (HClO) can prevent new infection by inactivating infectious viruses. Their virucidal effect is well recognized, and yet predicting the effects of oxidants on viruses is currently impossible because the detailed mechanisms of viral inactivation remain unclear. Our data show that ONOOH and HClO cross-linked the capsid proteins and RNA genomes of Qβ and MS2 phages. Consistently, the capsids appeared intact by transmission electron microscopy (TEM) even when 99% of the phages were inactivated by oxidation. Moreover, a precise molecular study of the capsid proteins shows that ONOOH and HClO preferentially targeted capsid protein regions containing the oxidant-sensitive amino acid C, Y, or W. Interestingly, the interaction of these amino acids was a crucial parameter defining whether they would be modified by the addition of O, Cl, or NO_2_ or whether it induced the loss of the protein region detected by mass spectrometry, together suggesting potential sites for cross-link formation. Together, these data show that HClO and ONOOH consistently target oxidant-sensitive amino acids regardless of the structural organization of Qβ and MS2, even though the phenotypes change as a function of the interaction with adjacent proteins/RNA. These data also indicate a potential novel mechanism of viral inactivation in which cross-linking may impair infectivity.

## Introduction

Water is a major vector for transmission of many human enteric viruses (e.g., noroviruses, hepatitis A and E viruses, enteroviruses). Transmission occurs either directly through consumption of drinking water or by swallowing water during recreational activities, or indirectly through consumption of food in contact with polluted water (i.e., lettuce, red fruits, shellfish) (Hirneisen et al., 2010; Blanco et al., 2017; Kauppinen and Miettinen, 2017; Hartard et al., 2019). Most enteric viruses are small particles (20–40 nm) composed of single-stranded RNA protected inside a capsid made of proteins. Inactivation of such viruses at each step of the water cycle is the key to limiting transmission to humans. A wide range of chemical oxidants naturally produced in waters or added during disinfection processes are recognized to have a virucidal effect (Shin and Sobsey, 2008; Cromeans et al., 2010; Page et al., 2010; Wigginton, K. et al., 2012; Wigginton, K. R. et al., 2012; Araud et al., 2018; Brié et al., 2018). Their effect has been largely demonstrated by using the cell culture approach, which is still considered to be the gold standard to evaluate virus inactivation. Nevertheless, the most important viruses for human health (i.e., noroviruses, hepatitis viruses) are still difficult to culture (Ettayebi et al., 2017; Fu et al., 2019). In-depth understanding of the mechanisms leading to viral inactivation would allow not only the fate of viruses to be predicted, especially those that are non-culturable or emergent, but also disinfection treatments to be optimized (Wigginton, K. et al., 2012). In addition, it would provide an explanation as to why viruses with similar structures show significant differences when exposed to disinfection treatments. Such challenges can be addressed today using a combination of biological (i.e., cell culture) and physico-chemical (i.e., protein mass spectrometry, structural virology approaches) techniques (Wigginton and Kohn, 2012).

Peroxynitrite (ONOOH) and hypochlorous acid (HClO) are two strong oxidants having a virucidal effect. Macrophages produce ONOOH which is highly reactive at physiological pH (pH 7.4) with aromatic cycles. It is produced during inflammation in humans, in whom the expression of inducible nitric oxide synthase (iNOS) is stimulated. iNOS forms ^∙^NO which combines with O_2_^∙–^, a superoxide ion produced by many sources including NADPH oxidase or mitochondrial respiration, to form ONOOH (Alvarez and Radi, 2003; Goldstein and Lind, 2005). At neutral and acidic pH, ONOOH splits into ^∙^OH and ^∙^NO_2_ which oxidize amino acids especially cysteine (C), methionine (M), tyrosine (Y), and tryptophan (W) residues (Alvarez and Radi, 2003). ONOOH was reported to inactivate viruses (Padalko et al., 2004; Yamashiro et al., 2018) but, to our knowledge, there is currently no consensus on the mechanism(s) by which it reacts with viruses. Two reports convincingly suggested that the inactivation of viruses by peroxidation was related to the formation of covalent bonds between capsid proteins and to the nitration of tyrosines (Meunier et al., 2004; Padalko et al., 2004). But, to our knowledge, the structural organization of viruses has not yet been addressed as involved in the inactivation of ONOOH-treated viruses.

HClO (E^0^ = 1.4 at pH 7.4) is widely used in industries for the disinfection of foodstuffs and drinking water because of its well-recognized anti-viral effect (Shin and Sobsey, 2008; Cromeans et al., 2010; Page et al., 2010; Wigginton, K. et al., 2012). Endogenously, HClO is produced by neutrophil granulocytes expressing myeloperoxidase in humans (Masuda et al., 2001). Mechanistically, there is a consensus that HClO oxidizes preferentially the thiol group of C and M residues (Pattison and Davies, 2001; Hawkins et al., 2003; Na and Olson, 2007). For other amino acids, the oxidation ranking order varies between studies and conditions, but Y residues are often cited as being significantly affected (Pattison and Davies, 2001; Hawkins et al., 2003; Bergt et al., 2004; Na and Olson, 2007). The chlorinated Y residue is therefore used as a marker of protein oxidation by HClO (Komaki et al., 2018). Moreover, HClO was shown to cause the formation of covalent bonds between amino acids and nucleotides *in vitro* (Hawkins et al., 2002; Hawkins and Davies, 2002). Amino acid modification and cross-linking may both result from a similar mechanism as there is evidence suggesting that HClO forms unstable chloramine with amine moieties of proteins; ^∙^Cl may then be transferred to neighboring amino acids until it encounters the side chain of Y residue where it may stabilize into chloro-tyrosine (Hawkins et al., 2003). For cross-linking, the exit of ^∙^Cl out of chloramine leaves N-centered radicals which may react further and create a covalent bond with an interacting molecule (Hawkins et al., 2002, 2003). However, to our knowledge, the formation of covalent bonds as a molecular phenotype has not yet been reported for the treatment and inactivation of viruses by HClO. Furthermore, at high concentrations HClO was also shown to induce protein breaks (Hawkins et al., 2003; Wigginton, K. et al., 2012). Therefore, as with ONOOH, there is currently no consensus on the mechanism(s) describing how HClO inactivates viruses. In addition, to our knowledge, the structural organization of viruses has not been fully addressed as involved in the inactivation of HClO-treated viruses.

F-specific RNA bacteriophages (e.g., MS2 and Qβ phages) belonging to the *Leviviridae* family are currently used as surrogates to express the behavior of pathogenic enteric viruses such as noroviruses in the environment or during disinfection treatments because of their “similar” sensitivity to oxidants and similar structural organization (Ogorzaly et al., 2009; Hartard et al., 2015, 2016, 2017). They replicate in the human gut microbiota, are transmitted through the fecal-oral route, and have similar structures and sizes (∼25 nm in diameter) compared to the main enteric viruses (Gorzelnik et al., 2016; Hartard et al., 2016; Dai et al., 2017). Qβ and MS2 phages are non-enveloped particles that contain single-stranded, positive-sense RNAs. Qβ displays three structural proteins (CP, A1, and A2) (Gorzelnik et al., 2016; Cui et al., 2017), whereas MS2 has two structural proteins (CP and A2) (Fiers et al., 1976; Dai et al., 2017). Their capsids are composed of 89 dimers of the major protein (CP), which are assembled in pentamers and hexamers to form a T3 icosahedral symmetry. One maturation protein is present in the capsid for both phages, which will be called “A2 protein” in this study for simplification purposes (Gorzelnik et al., 2016; Dai et al., 2017). Importantly, the A2 proteins which are in part located inside the capsids strongly interact with the RNA genome (Rumnieks and Tars, 2017). Only for Qβ phage, the A1 protein is present in 8–10 copies in place of some CPs. The differences in the numbers of “free” side chains of C (not forming disulfide bonds), M, and W residues (MS2 = 358 C, 363 M, 368 W; Qβ = 40 C, 23 M, 59 W) between the two phages suggest that they may be differentially sensitive to HClO and ONOOH even though they both display similar numbers of Y residues (MS2 = 728 Y, Qβ = 828 Y).

The aim of this study was to define the modifications at the capsid of two viruses (i.e., MS2 and Qβ phages) treated by two oxidants (i.e., ONOOH and HClO) targeting preferentially similar amino acids (i.e., C, M, W, and Y). Priority was given to the early modifications on the capsid proteins in the aim to determine potential mechanisms of viral inactivation. The results were mainly generated using transmission electron microscopy (TEM), SDS-PAGE, and mass spectrometry (MS), and the data were compared to studies showing the structural organization of these phages. The results show that HClO and ONOOH strike by targeting C, M, W, and Y amino acid residues of capsid proteins. Interestingly, oxidation leads to amino acid modifications (+O, +Cl, +NO_2_) or protein damage where sensitive amino acids are adjacent to another protein or the RNA. The compiled data suggest that one type of protein damage may be the formation of covalent bonds/cross-links as determined by SDS-PAGE. Taken together, this set of data suggest the mechanism by which oxidants inactivate viruses, and emphasizes the need for greater knowledge of the structural organization of viral particles to determine the mechanisms of inactivation by oxidation.

## Materials and Methods

### Production and Purification of Phage Suspension

**FIGURE 1:**
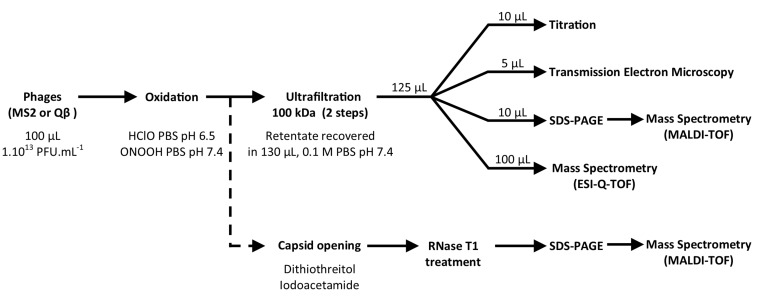
Experimental strategies to define the capsid modifications corresponding to viral inactivation by HClO and ONOOH.

Qβ and MS2 phage suspensions were prepared using a standard procedure previously described (Loison et al., 2016). Briefly, replication was conducted on *E. coli* K-12 Hfr ATCC 23631 as host cells in liquid culture maintained at 37°C with a rotational shaking of 150 rpm. Phages were inoculated with an M.O.I of 1 phage per 10 bacteria (assumed by measurement of absorbance at 600 nm) and the cultures carried on for 5 h. Suspensions were then centrifuged at 6000 g for 20 min at 4°C and filtered through by a Polyethersulfone (PES) membrane of 0.22 μm pore size to discard main cellular fragments and bacteria. Phages were concentrated by ultracentrifugation (21 krpm for 8 h at 4°C). Pellet were resuspended with 1.5 mL of 10 mM phosphate buffered saline (PBS) (137 mM NaCl, 2.7 mM KCl, 10 mM Na_2_HPO_4_, 1.8 mM KH_2_PO_4_, pH 7.4) and purified by ultracentrifugation (30 krpm for 18 h at 15°C) forming cesium chloride gradient density (0.63 g.mL^–1^ was used for both phages). Finally, cesium chloride was removed by two successive dialyses in bathes of 10 L of PBS 1 mM, pH 7.4 at 4°C. So purified phage suspensions contained 1 and 7.10^13^ PFU.mL^–1^ of Qβ and MS2 phages respectively. Stocks were diluted using 1 mM PBS, pH 7.4 to adjust concentrations to 1 × 10^13^ PFU.mL^–1^. Highly concentrated and purified phages were necessary to generate substantial data from SDS-PAGE, mass spectrometry and transmission electron microscopy. Absence of Qβ phage (or MS2 phage) in MS2 phage (or Qβ phage) suspension was verified by RT-PCR. Global procedures applied for oxidation and studying their effect is given in Figure 1.

### Inactivation of Phages by Oxidation

Two different oxidants were used to inactivate viruses: HClO and ONOOH. For HClO treatment: 1 volume (100 μL) of either Qβ or MS2 phages at 1 × 10^13^ PFU.mL^–1^ was treated with 1 volume of HClO:PBS 10 mM pH 6.5 (Fisher Scientific #10573094) diluted/prepared with pure water (DNase and RNase free). Phages were treated with the following final concentrations of HClO: 0, 670, 1346, and 2692 μM. Treatments were carried out during 10 min at 25°C in a thermomix with spinning at 600 rpm. This temperature was chosen to be close to environmental conditions. The pH of the reaction was measured at 6.5 and was chosen to favor the form HClO of the oxidant. Free chlorine equivalent of HClO solutions were controlled with DPD (N, N-diethyl-p-phenylenediamine) method, as described previously (Brié et al., 2018). The reactions were quenched by addition of sodium thiosulfate equimolar with the HClO. The same solutions were used but free of HClO for the controls which were processed simultaneously and in a similar manner as the samples containing HClO. As a reference for disinfection conditions, we suggest the CT (the disinfectant concentration (mg.L^–1^) multiplied by the contact time (min) with viruses) to be respectively of 0, 350, 700, and 1400 (min.mg.L^–1^), these CT values are based on the reaction occurring through the 10 min incubation, please note however most of the inactivation (>90%) occurred within the first 2 min, and no residual oxidant was observed at 10 min. The CT used in here to observe high levels of inactivation are therefore explained by the high concentration of phages contained in a small volume (by conversion 7.5 μg of HClO equivalent inactivated about 4–5 log_10_ of 10^12^ PFU at 25°C). Defining the concentrations of oxidants as the limiting factor was a choice based on obtaining better reproducibility for low levels of inactivation (<2 log_10_), this choice was further supported for being able to compare the inactivation phenotypes induced by HClO and ONOOH which is unstable timewise and therefore cannot be maintained at measurable concentrations, at pH of reaction (i.e., pH 7.4).

For ONOOH treatment: 1 volume (100 μL) of either Qβ or MS2 phages at 1 × 10^13^ PFU.mL^–1^ were added to 1 volume of 1 M KH_2_PO_4_ at pH 7.4 [pH at which ONOOH inactivated human pathogenic viruses (Padalko et al., 2004)] prior to two successive additions of 0.5 volume of ONOOH (Bertin #81565) due to the short half-life of peroxynitrite (Padalko et al., 2004) -ONOOH is pre-diluted in 0.3 M NaOH chilled in ice- to obtain the final concentrations of 0, 100, 800, and 4000 μM. The stock concentration of ONOOH was measured using spectrophotometric absorption at 302 nm with an epsilon 1670 M^–1^ cm^–1^, following manufacturer instructions. Because all ONOOH is consumed almost instantaneously in these conditions (pH 7.4), no quencher was used and no CT could be calculated. The incubation of this reaction ran for 30 sec and was then processed to the next step (see below). The controls without ONOOH were processed simultaneously and in a similar manner as the samples containing ONOOH.

Promptly (less than a minute) after either HClO or ONOOH oxidation treatments, the suspensions were submitted to two successive ultrafiltration steps on Amicon 100 kDa membrane using 10 mM PBS pH 7.4 to replace the former solvents/buffers/salts (Millipore #UFC510096, 13 krpm for 15 min). The potential loss of viral titer due to this step was measured for Qβ and MS2 phages, untreated by oxidants, and were lower than 0.5 log_10_. The retentate was resuspended in 130 μL of 10 mM PBS pH 7.4. This volume was then distributed for analysis of the remaining infectious phages (10 μL for titration), of the morphology of the capsid (5 μL for transmission electron microscopy or TEM), of the protein modifications (10 μL for SDS-PAGE ± MALDI-TOF MS) and of the amino acid modifications (100 μL for LC-ESI-QTOF) as shown in Figure 1. The all process was repeated three time carried out on independent days.

### Titration: Enumeration of Infectious Phages

Infectious Qβ or MS2 phages were quantified by using the standard double-layer agar method (NF EN ISO-10705-1 2001). Briefly, *E. coli* K-12 Hfr ATCC 23631 was used as host bacteria. Ten-fold dilutions were realized taking 10 μL initial volume after oxidation treatment. The plates were incubated for 18 h at 37°C prior to enumeration. The phage concentration was expressed in PFU. mL^–1^.

### Transmission Electron Microscopy (TEM)

Five microliter of Qβ or MS2 phages were used per sample for TEM observation. Grids were prepared as previously described (Loison et al., 2016; Robin et al., 2019). Briefly, negative staining was achieved by dropping the samples on carbon-coated grids. The droplet was then left in place for 180 s at room temperature and the excess liquid was discarded. The grids were then covered with 2% phosphotungstic acid (at the same pH as the sample) for 60 s and dried with filter paper. A CM200 transmission electron microscope (Philips, Andover, MA, United States) operated at 200 kV accelerating voltage was used to monitor the grids (Loison et al., 2016).

### SDS-PAGE and MALDI-TOF MS

Ten microliter of samples were diluted by 10 μL of sample buffer (120 mM Tris-HCl pH 6.8, 2.5% (w/v) SDS, 200 mM DTT, 15% (v/v) glycerol, 0.002% (w/v) bromophenol blue). The whole 20 μL were run on a SDS-PAGE as previously described (Loison et al., 2016). Briefly, 4% acrylamide stacking gel was placed on top of a 12% acrylamide gels, using a Mini-PROTEAN 3 cell set up (Biorad). For protein migration, tris-glycine buffer was used and 100 V was applied for 1h45. Precision Plus Protein (Biorad #1610363) was used as a standard size marker. Oriole (Biorad #1610496) was applied for 1 h to reveal protein bands. The images of the gel were captured by a Gel Doc system (Biorad).

Protein extraction from the gel for MALDI-TOF mass spectrometry analysis was inspired from two studies (Gundry et al., 2009; Da Silva et al., 2011) with some modifications. Briefly, each band of interest was excised with a scalpel and cut into ∼1 mm^3^ slices. Thirty microliter of CH_3_CN:100 mM NH_4_CO_3_ pH 8.0 (1v:1v) was added and incubated for 10 min at room temperature (destain). This solution was replaced with 30 μL of pure CH_3_CN and incubated for 10 min at room temperature (dehydration). The solution was replaced by 30 μL of pre-chilled 100 mM NH_4_CO_3_ pH 8.0 containing a total of 2.4 μg of trypsin (Sigma-Aldrich #T6567) and incubated for 30 min on ice. The excess of the solution containing trypsin was discarded and another ∼30–50 μL (enough to cover the dices of gels) of 100 mM NH_4_CO_3_ pH 8.0 was added. The samples were placed at 37°C for 18 h to allow trypsin digestion. Peptides were extracted by centrifugation at 12 krpm for 5 min with a bench centrifuge and addition of 20 μL of matrix (10 mg.mL^–1^) of α-Cyano-4 hydroxycinnamic acid (Sigma-Aldrich) prepared in (v/v) H_2_O 0.1% TFA/CH_3_CN).

For peptide analysis, 1 μL of the extracted fraction was spotted on an MTP 384 ground steel target plate and air dried. MALDI-TOF MS (Matrix-Assisted Laser Desorption Ionization – Time Of Flight Mass Spectrometry) analysis were performed on an Ultraflex III system (BRUKER Daltonik GmbH) equipped with a UV Nd:YAG 200 Hz laser (λ = 355 nm). The data were obtained in positive ion reflectron TOF mode (Da Silva et al., 2011). The resolution of the TOF analyzer was of an average of 15,000. Each spectrum represents the sum of 1,000 laser shots. External calibration was performed using MSCAL2 ProteoMass Peptide MALDI-MS (Sigma-Aldrich).

Specific capsid proteins peptide mass fingerprints were determined by mass spectrometry MALDI-TOF MS. Theoretical masses were obtained by theoretical digestion of capsid proteins of both phages using the online software: “PeptideMass” from “ExPaSy,” accrylamide adduct and up to one missed cleavage was considered. An inaccuracy of mass up to 20 ppm was tolerated. The sequences used as a reference were from the following accession numbers: “Qβ-CP”: UniProtKB/Swiss-Prot: P03615.2, “Qβ-A1”: GenBank: AEQ25549.1, “Qβ-A2”: GenBank: AEQ25544.1, “MS2-CP”: UniProtKB/Swiss-Prot: P03612.2, “MS2-A2”: GenBank: ABQ02459.1 Masses that were found in the blank (SDS-PAGE gel stained with Oriole free of proteins signal following the same protocol of extraction and trypsin digestion) and across proteins samples were disregarded as they would be peptide of autolyze of trypsin or human contaminants.

### LC-ESI-Q-TOF

Electrospray as a source was chosen to perform relative quantification in this study because nitration of tyrosine is unstable when using a MALDI (Sheeley et al., 2005). After oxidation treatments as described above, capsids were disrupted by reduction for 30 min at 37°C with the addition of 4 μL of dithiothreitol (DTT) (Sigma-Aldrich #43816) for a final concentration of 25 mM to open disulfide bridges of the capsid (Loison et al., 2016). Reduced cysteines were alkylated with the addition of 8 μL of iodoacetamide (Sigma-Aldrich #I6125) for a final concentration of 75 mM. It was followed by incubation for 1 h at 25°C away from light sources. Such treatment was enough to disrupt the capsid of Qβ but not that of MS2 having no disulfide bridges. Therefore, in the case of MS2 phages, 104 μL of 16 M urea was added at the reduction step for a final concentration of 8 M urea (in 0.1 M PBS pH 7.4). The samples were then washed twice in 50 mM NH_4_CO_3_ pH 8.0 for Qβ, and 50 mM NH_4_CO_3_ with 1 M urea pH 8.0 for MS2, using ultrafiltration Amicon 10 kDa (Millipore #UFC501096). The samples recovered in the retentate were resuspended for a final volume of 50 μL of the respective buffers. The capsid proteins were then digested by trypsin (Sigma-Aldrich #T6567) for 18 h at 37°C with an estimated molecular ratio of 1:20 (trypsin:substrate), the quantity of protein substrate per sample was estimated by the quantity of infectious phages (titration) or total particles (RT-qPCR) (Not shown).

Two microliter of samples was injected into a HPLC system (LC thermo/dionex U3000) with a C18-column [Acclaim PepMap 100 (1 mm X′ 150 mm – 3 μm and 100 A)]. The method was performed with a flow of 50 μL.min^–1^ at 40°C (oven). Solvent A (formic acid at 2% in MQ H_2_0) and solvent B (acetonitrile) were used in gradients as follows: *t* = 0 (5% of solvent B), *t* = 5 min (20% of solvent B), *t* = 30 min (42% of solvent B). In between sample injections, rinsing cycles were applied with 75% of solvent B. Prior to each injection, the baseline was set using 2% of solvent B. The HPLC system was coupled with ESI-Q-TOF mass spectrometry. HPLC provided the relative quantification aspect of the method and the ESI-Q-TOF provided the identity of each peak appearing on the chromatogram of the HPCL.

Mass spectrometry ESI-Q-TOF (Bruker micrOTOF-Q) was performed in positive ion mode. Source: 4500 V. The resolution of the quadrupole is 0.7 Da and 10,000 for the TOF. The validation of peptide identity was confirmed by fragmentation using CID with argon at 20 eV. Each run of sample was calibrated by ESI tuning mix (Agilent) in 10 mM of NH_4_HCO_3_.

For analysis, the theoretical masses of the peptides of the capsid proteins digested by trypsin by the online software “PeptideMass” from “ExPaSy,” zero to one miss cleavage were considered. One to four time positively charged peptides were considered, the corresponding m/z were calculated manually using the formula (m + n^∗^1.007)/n (“m” being the theoretical peptide mass and “n” the charged status). Only masses matching with a precision of less than 20 ppm with theoretical masses were considered. The identity of each peptide reported in this study was confirmed at least once using MSMS. The identities of peptides and amino acids modified by “+O, +Cl, +NO_2_” were also confirmed at least once by MSMS (for frequent modifications). Mass shifts used to search such modifications were respectively “+15.9949 (+O), +33.9610 (+Cl -H), +44.9850 (+NO_2_ -H).” The proportion of one amino acid residue being modified by “+O, +Cl, +NO_2_” (as shown in Figure 4) was calculated based on the surfaces of the peaks of the related peptides, observed on chromatograms provided by the HPLC coupled with ESI-Q-TOF (surface peak of modified peptide “A”/(surface peak of peptide “A” unmodified + surface peak of peptide “A” modified)^∗^100), as observed in Supplementary Figure S1. Data analysis 4.2 software (Bruker) was used for spectrum analysis. The protein coverage were ∼21–28% for Qβ-A2, ∼35% for MS2-A2, 51% for MS2-CP and 93% for Qβ-CP.

### Capsid Opening for RNA Digestion

Thirty microliter of Qβ phages (initial concentration of 1 × 10^13^ PFU.mL^–1^) was treated with ONOOH at 800 μM using the same protocol as in the oxidation section of this manuscript. After oxidation treatments, capsid of Qβ phages were disrupted by reduction (DTT + iodoacetamide) as described above. One microliter of RNase T1 (Thermo Fischer Scientific #EN0541) was added per sample and the samples were incubated at 37°C for 2 h, following manufacturer instructions. They were then washed with a final concentration of 1 M NaCl to break RNA-protein non-covalent bond interactions and re-concentrated using Amicon (Millipore #UFC510096) for a final volume of 20 μL. The samples were diluted using sample buffer at a final concentration of 0.06 M Tris HCl pH 6.8, 8 M urea, 100 mM DTT, 1.25% (w/v) SDS, 7.5% (v/v) glycerol, and 0.002% (w/v) bromophenol blue prior to be resolved on 12% SDS-PAGE gels in tris-glycine buffer (as described here above). The gels were revealed using Oriole according to the manufacturer’s instructions and the pictures captured by a Gel Doc system (Biorad).

### Data Analysis

Unless specified, error bars in graphics indicate standard errors (SEM). All statistical analyses were performed using R statistical software (Rx64 v.3.5.3). The Shapiro-Wilk test was performed to check normality of the data with alpha level of 0.05. If data set followed a normal distribution (*p* > 0.05), parametric tests were applied. A paired or unpaired sample Student’s *t*-test was performed for dependent or independent data following a normal distribution, respectively. Non-parametric tests were used for data sets having a non-normal distribution (*p* < 0.05). For dependent or independent data with non-normal distribution, a Wilcoxon signed-rank test or a Mann–Whitney *U*-test was applied. For all tests, the significance level was set to 0.05.

## Results

### Inactivation of Qβ and MS2 Phages by HClO and ONOOH

**FIGURE 2:**
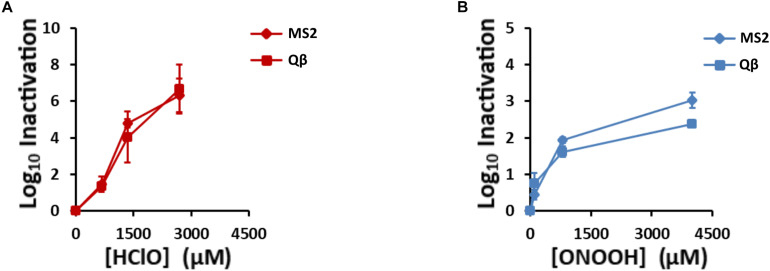
HClO and ONOOH inactivate Qβ and MS2 phages. Qβ and MS2 inactivation levels increase with **(A)**, HClO and **(B)**, ONOOH concentrations. HClO- and ONOOH-induced inactivation curves are plotted in function of the concentrations of the oxidant. Diamonds and squares markers stand for MS2 and Qβ phages, respectively. Each point on the curves is the result of at least three independent experiments carried out on separate days. Error bars stand for SEM.

Knowing the virucidal activity of HClO and ONOOH, we expected that they would inactivate Qβ and MS2 phages. Indeed, their inactivation was clearly observed for different concentrations of HClO and ONOOH (Figures 2A,B). Interestingly, although HClO and ONOOH induced different levels of inactivation, both phages were inactivated to a similar extent by both oxidants. For concentrations lower than 800 μM, the two oxidants caused similar inactivation of Qβ and MS2 phages. Indeed, treatments of 800 μM of ONOOH and 670 μM of HClO inactivated 1.6 and 1.3 log_10_ units of Qβ phages and 1.9 and 1.5 log_10_ units of MS2 phages, respectively. On the other hand, at higher concentrations, ONOOH had a limited additional effect on phage inactivation, whereas HClO allowed a gradual effect on phage inactivation as a function of concentration (Figures 2A,B). The phenotype difference between the two oxidants may be explained by the ability of ONOOH to self-react and decompose (Goldstein and Lind, 2005). Higher concentrations of ONOOH may therefore have promoted self-reaction rather than reaction with Qβ and MS2 phages. Nevertheless, together, these data show that both ONOOH and HClO were able to inactivate more than 99% of large numbers of both phages (100 μL of 10^13^ PFU.mL^–1^), and therefore that they can be used to study the mechanisms of viral inactivation by oxidants. In order to focus on the early modifications occurring during inactivation by oxidation, special attention was given to phenotypes appearing within the first two log_10_ of phage inactivation.

### Morphology of Inactivated Phages by TEM Observation

**FIGURE 3:**
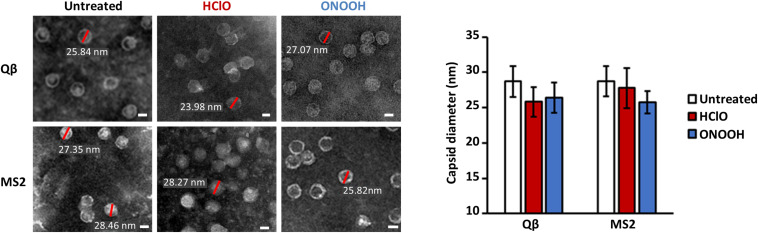
HClO and ONOOH inactivate Qβ and MS2 phages without dissolving their capsids. Left panel shows transmission electron microscopy pictures of Qβ and MS2 phages with and without inactivation by HClO and ONOOH, respectively; the pictures are representative of between 1 and 3 log_10_ units of inactivation. Sizes of capsids corresponding to red bars are written in white. Scale bars correspond to ∼20 nm and samples were observed with the magnification X 80,000. The pictures are representative of at least two independent experiments carried out on separate days. The mean capsid diameters of Qβ and MS2 phages in the conditions displayed on the left panel, as observed by transmission electron microscopy, are reported on the right panel. The means capsid diameters are the results of twenty measures per condition. Error bars stand for standard deviation.

In the scale of the first two log_10_ of phage inactivation, the capsids appeared unmodified by TEM (Figure 3). As previously observed, untreated Qβ and MS2 phages appeared as circles of about 27–28 nm of diameter in average (Figure 3; Dika et al., 2011; Loison et al., 2016). Interestingly, inactivated Qβ and MS2 phages by HClO and ONOOH still appeared clearly (Figure 3), on the other hand the average diameters of their capsids reduced slightly (25–27 nm in average) compared to when untreated (Figure 3). Together, these data strongly suggest that the initial mechanism(s) of Qβ and MS2 phage inactivation by ONOOH and HClO do(es) not rely on capsid dissolution but instead would be more related to molecular modifications.

**FIGURE 4:**
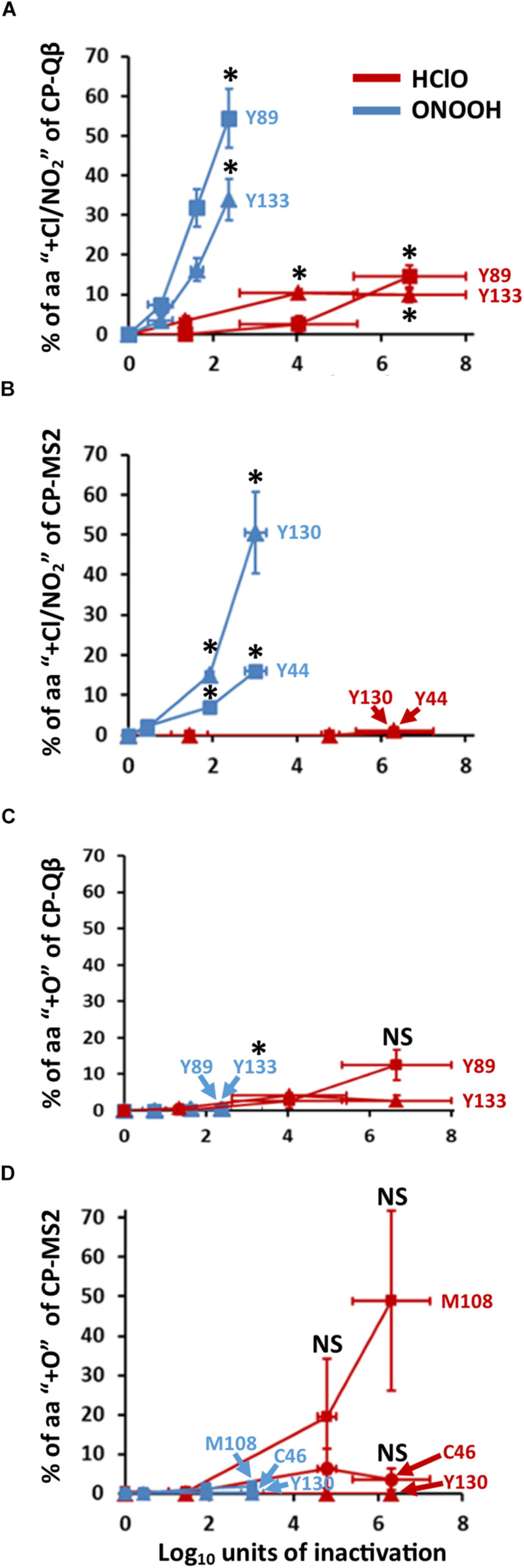
HClO and ONOOH stably oxidize C, M, Y, W. The curves represent the percentage of indicated residues which are stably oxidized. The addition of chloride adducts -for HClO treatment- and nitration -for ONOOH treatment- is represented in **(A)** for Qβ, and **(B)** for MS2. The addition of oxygen atoms is represented in **(C)** for Qβ, and **(D)** for MS2. The curves in blue are for ONOOH and those in red are for HClO. The identities of the residues of CPs which were modified are indicated accordingly. Each point of the curves is representative of three independent experiments carried out in separate days. Error bars stand for SEM. Where indicated *p < 0.05 as compared to control, the statistical tests are described in “Materials and Methods” section of this manuscript.

### Amino Acid Modifications on Phage Capsids After Inactivation by Oxidants

Modifications on the CPs of both Qβ and MS2 were defined using LC-ESI-Q-TOF-MS, MSMS (Figure 4). ONOOH caused significant nitration mainly of Y residues (Supplementary Figures S1–S3), with up to 30% of some Y nitrated for levels of inactivation lower than 2 log_10_ (Figures 4A,B). It peaked at 55% for Y89 of Qβ-CP at 2.4 log_10_ of inactivation and 50% for Y130 of MS2-CP at 3.0 log_10_ of inactivation. Infrequent (≤2%) nitration of W residues was also detected (not shown) and ONOOH induced the oxidation (+O) of C, M, and Y residues in low proportion (≤2%) regardless of the level of inactivation and the phage (Figures 4C,D).

HClO also modified amino acid residues in the CPs of both phages, but on a rather infrequent basis (≤7%) and only for one amino acid of Qβ (Y89) at levels of inactivation lower than 2 log_10_ (Figure 4). This was unexpected given the propensity of HClO to inactivate Qβ and MS2 under our experimental conditions (Figure 2A). Nevertheless, some amino acids were frequently modified by HClO at higher levels of inactivation. About 50% of M108 of MS2-CP were found to be oxidized (+O) at 6.3 log_10_ of inactivation (Figure 4D), and ∼10% of Y89 of Qβ-CP were found to be chlorinated (+Cl) (Figure 4A) and oxidized (+O) (Figure 4C) at 6.7 log_10_ of inactivation.

Together, these data show that oxidation of C, M, and Y amino acid residues of capsid proteins defines only highly inactivated viruses (≥2 log_10_) for HClO treatments, whereas oxidation of C, M, Y, and W amino acid residues of capsid proteins defines both low and highly inactivated viruses for ONOOH treatments. Overall, these results were intriguing because HClO and ONOOH oxidized the same amino acid residues for both phages (Figure 4) and several other C, M, Y, and W sensitive amino acids were detected by mass spectrometry but not found to be oxidized even at high levels of inactivation. Indeed, Y63, Y66 of Qβ-CP and Y58 of MS2-CP were not found to be oxidized. Therefore, we further questioned the potential fate of sensitive amino acid residues of phage by oxidation.

### HClO and ONOOH Target Protein Regions/Peptides Containing Sensitive Amino Acids

In order to investigate the phenotype difference between sensitive amino acids, we first observed the potential loss/damage of CP regions of Qβ and MS2 phages as a function of the levels of inactivation induced by oxidation, thinking that such loss could reflect potential cleavage or cross-linking. These protein regions were defined by trypsin digestion yielding predictable peptides, quantified by C18 liquid chromatography coupled with ESI-Q-TOF-MS, and their identities confirmed by MSMS (see section Materials and Methods). Using this approach, we observed that HClO and ONOOH interestingly affected CP regions with great heterogeneity (Supplementary Figure S4). Indeed, some CP regions were not found to vary between levels of inactivation, whereas others lost up to 15% of their intensity per log_10_ of inactivation (Supplementary Figure S4C), starting at levels of inactivation lower than 2 log_10_. Namely, the most affected peptides of Qβ- and MS2- CPs by ONOOH were 26–47 and 45–50, respectively, as compared to the least or not affected peptides 69–87 and 40–44, respectively. Similarly, the most affected peptides of Qβ- and MS2- CPs by HClO were 18–25 and 45–50, respectively, as compared to the least or not affected peptides 111–133 and 115–130, respectively (Supplementary Figure S4). Consistent with the literature (Wigginton and Kohn, 2012), we observed that the peptide 45–50 of MS2-CP was the most affected by HClO (Supplementary Figure S4D), and this was also true with ONOOH (Supplementary Figure S4C). Interestingly, this peptide contains a sensitive amino acid, C47. Therefore, we wondered if most damaged protein regions had sensitive amino acids. To test this possibility, we focused on the maturation proteins of Qβ and MS2 for their unique presence in each phage particle and therefore specificity of result. Indeed, we found that regions containing C, M, Y, or W were more likely to be preferentially affected. Considering all Qβ- and MS2- A2s with both HClO and ONOOH treatments, 70% of 17 peptides containing C, Y, and W were greatly affected compared to 19% of 26 peptides free of sensitive amino acids (Table 1).

**TABLE 1:** HClO and ONOOH preferentially affect peptides containing C, Y, W. Case of protein A2-Qβ and -MS2.

	% Affected (n)	% Unaffected (n)	n total
Having CYW	70 (12)	29 (5)	17
Missing CYW	19 (5)	81 (21)	26

*Results based on Supplementary Figure S8 and Supplementary Table S2.*

### HClO and ONOOH Cross-Link the Capsid Proteins and RNA Genomes of Qβ and MS2

**FIGURE 5:**
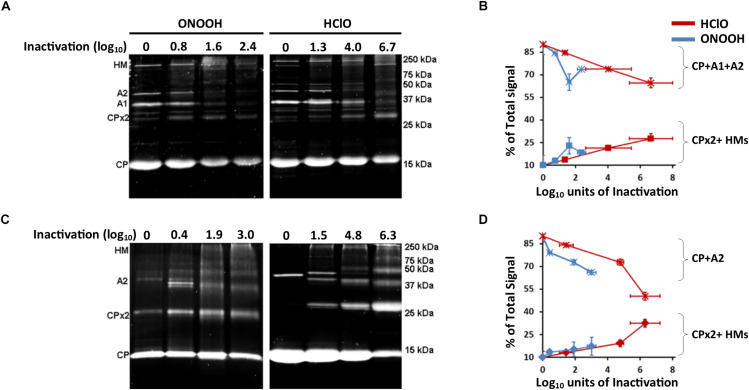
HClO and ONOOH cross-link Qβ and MS2 capsid proteins. **(A)** Images of SDS-PAGE stained with Oriole showing the capsid proteins masses of Qβ phage when inactivated or not by ONOOH- in the left panel and HClO- in the right panel, the levels of inactivation are indicated on top the gels. Capsid protein names are showed on the left side of the gels, markers of size are indicated on the right side of the gels. **(B)** Curves showing the evolution of the constitution of Qβ phage capsid proteins by the treatment of HClO- and ONOOH- induced inactivation as observed by SDS-PAGE; the star marker is for the added percent intensities of single proteins CP + A1 + A2, the square marker is for the added percent intensities of cross-linked proteins CP dimer (CPx2) + CP-A1-A2 multimer (HMs). **(C)** Image of SDS-PAGE stained with Oriole showing the capsid proteins masses of MS2 phage when inactivated or not by ONOOH- in the left panel and HClO- in the right panel, the levels of inactivation are indicated on top the gels. Capsid protein names are showed on the left side of the gels, markers of size are indicated on the right side of the gels. **(D)** Curves showing the evolution of the constitution of MS2 phage capsid proteins constitution by the treatment of HClO- and ONOOH- induced inactivation as observed by SDS-PAGE; the star marker is for the added percent intensities of single proteins CP + A2 proteins, the square marker is for the added percent intensities of cross-linked proteins CP dimer (CPx2) + CP-A2 multimer (HMs). In **(A–D)**, each result is representative of at least three independent experiments carried out on separate days. Error bars stand for SEM.

Oxidants including HClO and ONOOH were previously reported to induce cross-linking between proteins or cleavage of proteins. Such modifications in a complex molecular organization such as phages were therefore investigated here by SDS-PAGE on the grounds that protein breakage would lead to lighter proteins migrating further in the gel, and protein cross-linking would lead to heavier proteins and reduced migration. However, sometimes proteins can appear heavier by interacting with other molecules, therefore samples were treated with chaotropes such as urea to link such a phenotype to covalent bonding only. First, without any oxidation treatment, the proteins of Qβ and MS2 phages appeared at their expected masses (Figures 5A,C), and protein identities were confirmed by MALDI-TOF MS (Supplementary Figures S5, S6 and Supplementary Table S1). Interestingly, both HClO and ONOOH induced the appearance of heavier proteins, as observed by SDS-PAGE, suggesting that they induced protein cross-linking for both phages. Indeed, we were able to determine that they induced the formation of dimers (CPx2) and multimers (A1-A2-CPs for Qβ and A2-CPs for MS2) by observing the masses of the proteins by SDS-PAGE and by performing protein identification by MALDI-TOF MS (Supplementary Table S1). Importantly, the proportions of signal intensities of cross-linked proteins increased with the inactivation levels and the loss of signal for all capsid proteins at their original masses (Figures 5B,D and Supplementary Table S1). Altogether, these data show that oxidation by HClO and ONOOH of Qβ and MS2 phages correlated with the levels of protein cross-linking and inactivation.

Oxidants also cross-link proteins to RNA (Mirzaei and Regnier, 2006; Poria and Ray, 2017). Therefore, we hypothesized that it could occur with viral particles as well. This was investigated by suggesting that RNA hydrolysis would rescue protein intensities at their original masses as observed by SDS-PAGE. And indeed, by hydrolyzing RNA with RNase T1, A1 proteins of Qβ phages, whose intensity sharply decreased after 800 μM ONOOH treatment, were partially recovered (Supplementary Figure S7A). Moreover, because the samples were washed with 1 M NaCl, a procedure which is used to separate proteins and RNAs interacting with non-covalent bonds (Athavale et al., 2010), it further supports that ONOOH induced the formation of covalent bonds between A1 and the RNA genome. The identity of the A1 protein was confirmed in each sample by MALDI-TOF MS (Supplementary Figures S7B,C). By contrast, the original intensity of Qβ-A2 was not rescued after RNase treatment. It suggested that 800 μM ONOOH treatment may have bound Qβ-A2 to other neighboring proteins but not or not only to RNA. Together, these data strongly support that ONOOH treatment also induced the formation of covalent bonds between proteins and RNA at least for Qβ phage.

Based on these results, we wondered if interaction between molecules changed the modifications of capsid protein regions containing sensitive amino acids, induced by HClO or ONOOH. Indeed, it was a decisive parameter. By comparing the data to the molecular organization of both phages as reported in the literature (Gorzelnik et al., 2016; Cui et al., 2017; Dai et al., 2017; Rumnieks and Tars, 2017) or with protein data bank (PDB) files, we observed that the regions mostly affected were interacting regions. More specifically, the peptides mostly affected by oxidants were found to interact with neighboring proteins or RNA genomes, and it was especially true for protein regions containing sensitive amino acids. Indeed, considering the A2 proteins of both phages, only 14% of the interacting peptides missing C, Y, W residues (directly or indirectly) were preferentially affected. By contrast, 88% of the peptides were greatly affected when they interacted with C, Y, W residues (Table 2 and Supplementary Table S2). This can be illustrated as follows for MS2 phage: HClO and ONOOH mostly affected CP-45-50 (Supplementary Figures S4C,D), of which the C47 residue is located between T46 and S48 both interacting with at least 16 RNA hairpins (Dai et al., 2017). Both oxidants strongly damaged A2-51-62 having Y60 as close as 2.7 Å to the RNA genome (Supplementary Figures S8C,D,F, S9A,B) (Dai et al., 2017). The posterior region of MS2-A2 was also particularly damaged by both oxidants, of which the peptide 359-372 with Y370 interact with an adjacent CP (Supplementary Figures S8C,D,F, S9A,C, S10). Similarly for Qβ phage, the posterior region of A2 was reported to be in close interaction with CPs (Cui et al., 2017) (Supplementary Figure 11). And, HClO strongly affected A2-125-135 which interacts with Y133 of an adjacent CP (Supplementary Figures S8A,E, S11A,B). The latter finding suggests that, regarding the sensitivity to oxidants, interacting with an adjacent C, M, Y, W equates to containing a sensitive amino acid. Notably, no peptides containing solely an M as a sensitive amino acid were found to be preferentially affected, thus suggesting that the residues of interest may be C, Y, W rather than C, M, Y, W.

**TABLE 2:** HClO and ONOOH preferentially affect peptides in interaction with C, Y, or W. Case of protein A2-Qβ and -MS2.

		% Affected (n)	% Unaffected (n)	n total
Interact	Missing CYW	14 (1)	86 (6)	7
	With CYW	88 (7)	13 (1)	8
Do not interact	Missing CYW	21 (4)	79 (15)	19
	Having CYW	56 (5)	44 (4)	9

*Results based on Supplementary Figure S8 and Supplementary Table S2.*

The peptides free of interaction with neighboring proteins or RNA genomes were less likely to be greatly affected. Indeed, only 32% of the non-interacting peptides were found to be strongly affected by oxidant treatments (Table 2). Notably, neither HClO nor ONOOH treatments greatly affected the 4–9 and 3–19 peptides of the A2 proteins of Qβ and MS2 phages, respectively (Supplementary Figures S8A–D), which are both non-interacting peptides (Gorzelnik et al., 2016; Cui et al., 2017; Dai et al., 2017; Rumnieks and Tars, 2017). This was also true even for peptides directed toward the outside of the capsid supposedly very accessible to solvents/oxidants. Indeed, 2–14 of Qβ-CP formed a protruding loop on the outer side of the capsid (Supplementary Figure S12A), 111–133 of Qβ-CP was also located on the outer side of the capsid (Supplementary Figure S12B) and both were minimally affected (Supplementary Figure S4A).

Finally, nearly all the amino acids modified by the addition of O, Cl, or NO_2_ (Figure 4) were not found to be interacting. From this observation, we can assume that these types of modification are predictable for non-interacting sensitive amino acids, even though not all of them were observed to be oxidized, under these experimental conditions. Conversely, nearly all peptides containing sensitive amino acids at interaction sites were found to be preferentially affected by HClO and ONOOH. Therefore, following this pattern, we concluded that the structural organization of Qβ and MS2 phages affected the molecular modifications induced by HClO and ONOOH. Altogether, these data also pointed toward the formation of cross-links as potential mechanism for viral inactivation by oxidation, under these experimental conditions.

## Discussion

Considering that HClO and ONOOH inactivate viruses in the environment during disinfection processes or in humans during inflammation, this study was conducted to determine the molecular modifications occurring during their inactivation. Such knowledge is the key to predicting inactivation of viruses that are difficult to culture in laboratory or emerging. The existing literature on the topic indicates that inactivation may be linked to the modification of sensitive amino acids, backbone cleavage or cross-linking of structural molecules (Padalko et al., 2004; Wigginton, K. et al., 2012). Nucleic acid should be less affected because the constant rates of some amino acids are several orders of magnitude higher than those of nucleotides (Wigginton and Kohn, 2012). We therefore chose to focus on capsid modifications, keeping in mind the results of another study stating that HClO affects without doubt the capsid but also surprisingly genome replication (Wigginton, K. et al., 2012).

The oxidations of single amino acids and nucleic acids have been thoroughly studied. HClO is known for oxidizing sulfhydryl-containing amino acids, i.e., C and M. HClO can also oxidize the alpha amino group of amino acids and the side chains of H, K, W, Y, and R (Hawkins et al., 2003), these sensitive amino acids have therefore been considered in this study, however the low abundance and the absence of H at interaction site on the detected peptides, in both Qβ and MS2 phages, prevented us to conclude on the potential implication of H. Regarding nucleic acids, the product of the reaction with HClO mostly leads to the formation of chloride adducts on the RNA bases (Masuda et al., 2001). ONOOH is well-known for nitrating Y and W but it can also oxidize C and M (Alvarez and Radi, 2003; Radi, 2018). It also nitrates RNA bases such as guanosines and adenosines (Masuda et al., 2002). Using mass spectrometry, we were able to confirm the effects of these two oxidants. Notably, HClO oxidized (+O) C, (+O) M, and (+O, +Cl) Y. Interestingly, HClO induced the addition of chloride adducts mostly only on the Y of Qβ phages. It is surprising because MS2 phages display conserved Y compared to Qβ phages, (Y130 of MS2-CP and Y133 of Qβ-CP). This shows that predicting the outcome of an oxidant reaction with a complex molecular organization such as that of viral particles is not straightforward. In the context of proteins, halogenation of the amine group is unstable, which leads to its dissociation with the chloride radical moving onto other amino acids until it reaches Y where it may stabilize in position 3 (Hawkins et al., 2003). Therefore, HClO may add chloride adducts to Y directly or indirectly. By dissociating with the amine group, there remains a N-centered radical that might recombine and form a covalent bond with neighboring molecules, as determined by EPR (Hawkins et al., 2002, 2003). For ONOOH, dimerization by oxidation reactions of proteins or nucleic acids has also been well described. Indeed, the dimerization of nucleic acids has been previously reported (Yun et al., 2011) as well as it was described for proteins by the formation of di-tyrosine covalent bonds (Alvarez and Radi, 2003). Thus, both oxidants seem to be able to stably oxidize amino acids by the addition of +O, +Cl, or +NO_2_, but they also oxidize proteins and nucleic acids in a less stable manner, i.e., cross-linking molecules (Hawkins et al., 2002; Hawkins and Davies, 2002; Alvarez and Radi, 2003; Meunier et al., 2004; Padalko et al., 2004; Yun et al., 2011). So far, our data are consistent with the phenotypes that have previously been observed after HClO or ONOOH treatment. Moreover, our data strongly suggest that the unstable/cross-linking oxidation would occur first for Qβ and MS2 phages. Indeed, substantial formation of dimers/multimers between capsid proteins for both phages was observed with both oxidants and from the early stages of inactivation, cross-linking between capsid proteins and RNA genomes for Qβ phages was also observed when less than 2 log_10_ were inactivated by ONOOH. Therefore, we propose that the dimerization/multimerization process is the main mechanism by which HClO and ONOOH inactivated Qβ and MS2 phages.

For Qβ and MS2 phages, the genome is injected through the F-pili of bacteria (Paranchych et al., 1971). So, eventually the formation of cross-links between capsid proteins and RNA genomes would prevent genome injection and thus bacterial infection. The second possibility could be related to the covalent binding of proteins to RNA genomes which would prevent their translation once in the host bacteria. So far, these hypotheses are consistent with previous reports in which 50% of phages inactivation by HClO was caused by preventing the injection of the RNA genome into the bacterial host cell and the other 50% by preventing its translation (Wigginton, K. et al., 2012). The latter explanation could also be linked to the direct modification of the RNA genome by HClO. Then, Padalko et al. (2004) showed that ONOOH cross-linked the CPs of Coxsackievirus CBV3, which were also nitrated as determined by western blotting (Padalko et al., 2004). Interestingly, the same authors reported that ONOOH prevented the injection of the RNA genome into the host cell but not the binding of the virus to its receptor (Padalko et al., 2004). Importantly, neither HClO nor ONOOH prevented recognition of the cell receptor by the virus, and both of them prevented injection of the RNA genome into the host cell, which together support that these two oxidants inactivate viruses by a similar mechanism (Padalko et al., 2004; Wigginton, K. et al., 2012). In our opinion, only ONOOH induced sensitive amino acid oxidation with the addition of “O” or “NO_2_” in the CPs of Qβ and MS2 phages at low levels of inactivation but both ONOOH and HClO promoted the formation of cross-links, which supports that it would be the main mechanism of phage inactivation at the earliest stages.

As the dimerization/multimerization process seems to occur first at the earliest stages of phage inactivation, it interestingly suggests that C, Y, and W residues will be more sensitive to oxidants if they are in interaction with a neighboring capsid protein or the RNA genome. This emphasizes that the electrostatics modifying their electronic features may promote the instability of C, Y, and W oxidation. Further investigations are necessary to better predict the effects of oxidants on the complex molecular organization of viral particles. Nevertheless, the present study shows that the peptides primarily damaged by HClO and ONOOH can differ. It is probably related to their respective intrinsic reactivities with amino acids and nucleic acids accordingly to the local pKa, which may vary with the local environment depending, in part, on the interacting molecule (Isom et al., 2011; Thaplyal and Bevilacqua, 2014). Further investigations are also needed to better understand the mechanism by which oxidants inactivate Qβ and MS2 phages focusing on cross-link formation for the prevention of phage infectivity as a function of the oxidant used.

As regards to the techniques and protocols used in the present study, our results are consistent with other authors who reported a similar decline in peptide intensity for MS2 treated with HClO (Wigginton and Kohn, 2012), especially for the CP peptide 45-50,which was the most affected in both works. For the A2 protein, we observed again high consistency with their work considering that peptides 90–99, 132–147, 148–162, and 220–229 were among the least affected by HClO treatment. Therefore, we are confident in the phenotypes reported here for ONOOH treatment and for Qβ. On the other hand, Wigginton et al. suggested that the decrease in the detection of peptide 45–50 resulted from its cleavage (up to ∼30–40%), whereas our data would suggest otherwise because we did not observe CP breakage under any of the tested conditions. This discrepancy may stem from the difference in the principles of the techniques used. Indeed, Wigginton et al. used MALDI type of source of mass spectrometer and observed protein cleavage, whereas we used SDS-PAGE and observed protein cross-linking. Nevertheless, it is healthy to consider that CP-45-50 of MS2 could break under HClO treatment, if CP is cross-linked at other interaction sights then cleavage would not necessarily be apparent by SDS-PAGE. Further investigation may be necessary to clarify the modifications induced by HClO on this specific peptide.

## Conclusion

In conclusion, the results of this study show that HClO and ONOOH affect sensitive amino acids (C, M, Y, W), which may result in their modification (+O, +Cl, or +NO_2_) or possible cross-linking depending on the presence of adjacent proteins/RNA. Altogether, these findings could provide novel diagnostic tools for pathogenic viruses in food industries and the development of disinfection models for aqueous media to predict the level of inactivation of single-stranded RNA viruses by oxidants such as HClO and ONOOH.

## Data Availability Statement

All datasets generated for this study are included in the article/Supplementary Material.

## Author Contributions

GB wrote and edited the manuscript, designed experiments, performed SDS-PAGE and MALDI-TOF, prepared samples for TEM and LC-ESI-Q-TOF, analyzed the data of LC-ESI-Q-TOF, and generated the captures of PDB files. PL developed (i) the SDS-PAGE conditions to study viruses in reduced conditions, (ii) TEM, and (iii) the protocol for sample preparation for MALDI-TOF. LV-L was intellectually involved in the development of method for MALDI-TOF and actively participated to their generation. FD performed analysis by LC-ESI-Q-TOF as well as the MS and MSMS spectrum analysis. JC provided highly pure and highly concentrated Qβ and MS2 suspensions. DM, NB, GK, and CG were intellectually involved in the development of the project and funding acquisition. CG also participated in the experimental design, interpretation of experimental data, and co-wrote the manuscript. All authors contributed to manuscript edition and approved the submitted version.

## Conflict of Interest

The authors declare that the research was conducted in the absence of any commercial or financial relationships that could be construed as a potential conflict of interest.
