# Supplementary material for: Structural Organizations of Qβ and MS2 Phages Affect Capsid Protein Modifications by Oxidants Hypochlorous Acid and Peroxynitrite
Source: Front Microbiol. 2020 Jun 3;11:1157. doi: 10.3389/fmicb.2020.01157 (PMC7283501; doi:10.3389/fmicb.2020.01157)
Supplement: Supplementary file 1 [file Data_Sheet_1.pdf]

Supplemental Figure 1

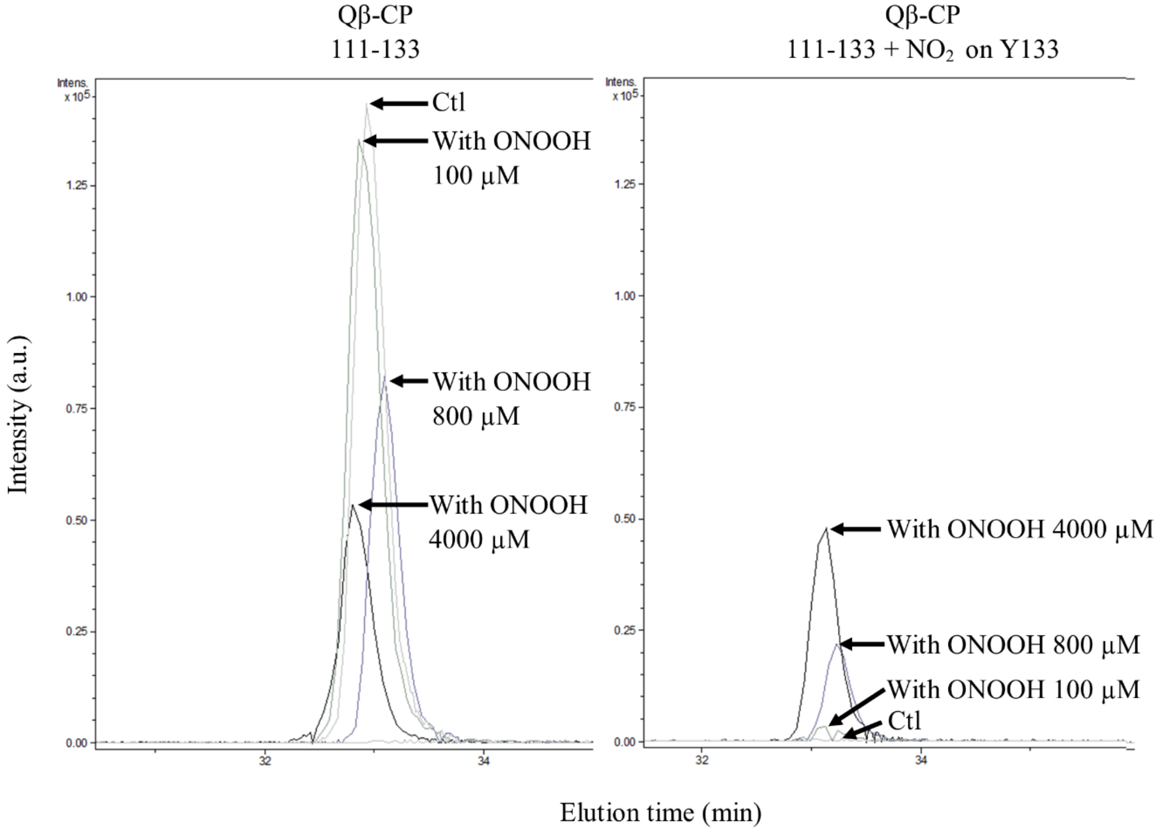

<u>Supplemental figure 1</u>: HPLC chromatograms of HPLC showing peak intensities of unmodified (left side) and nitrated (right side) peptide 111-133 of Qβ-CP. The concentrations used to treat Qβ phages to obtain the shown signals are as indicated by black arrows. These data are representative of one independent experiment and have been reproduced in three independent experiments carried out in separate days.

Supplemental Figure 2

(A)

Qβ-CP Nitration of Y-133 by ONOOH Nitration mass is (NO_2) 45,992903- (H)1,007825 = 44.985078 Da

Qβ-CP: 111-TELAALLASPLLIDAIDQLNPAY-133

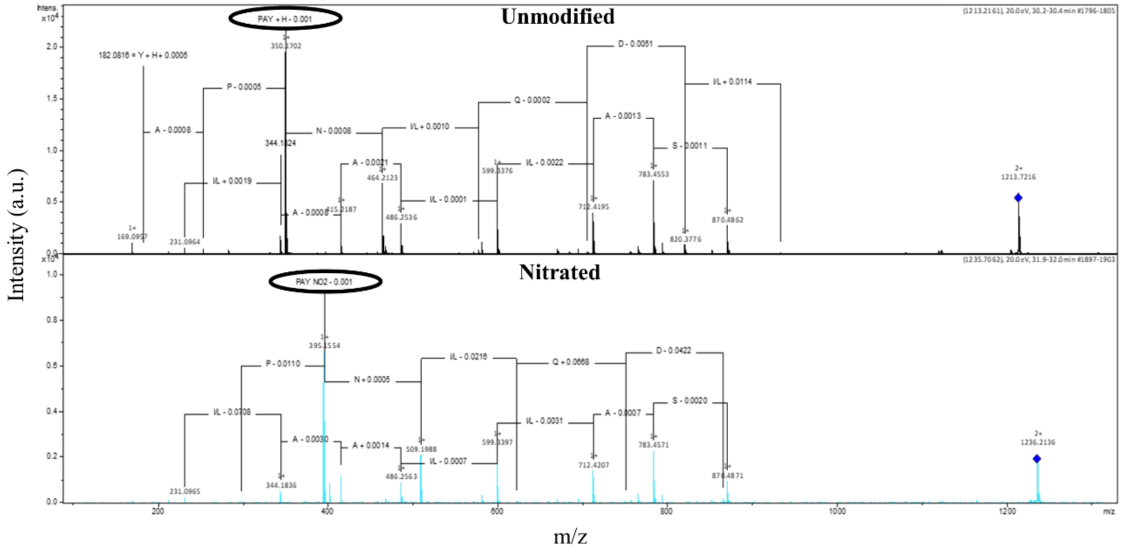

(B)

Qβ-CP Nitration of Y-89 by ONOOH Nitration mass is (NO_2) 45,992903- (H)1,007825 = 44.985078 Da

Qβ-CP, peptide: 87- QAYADVTFSFTQYSTDEER -105

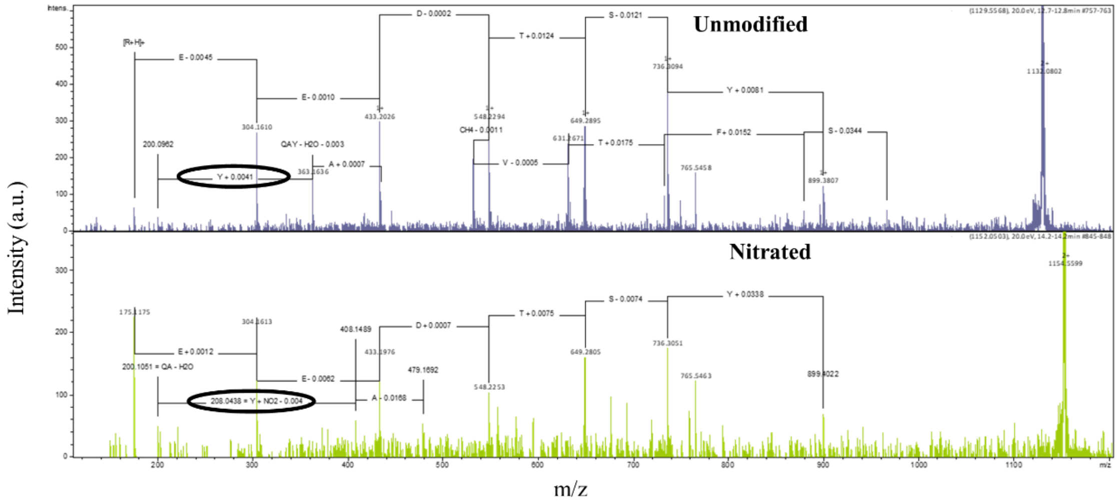

Supplemental figure 2: LC-ESI-Q-TOF and CID generated MSMS spectra of unmodified (upper panels) and nitrated (lower panels) Qβ-CPs peptides containing in **A**, -Y133 and in **B**, -Y89. The sequence of each peptide is indicated above respective panels. Masses differences between peaks have been annotated with corresponding amino acids (in one letter code), beside the suggested identity of amino acids are indicated the difference of mass considered as compared to the theoretical masses of the indicated amino acid.

Supplemental Figure 3

(A)

MS2-CP Nitration of Y-130 by ONOOH   Nitration mass is (NO_2) 45,992903- (H)1,007825 = 44.985078 Da

MS2-CP, peptide: 115- DGNPIPSAIAANSGIY -130

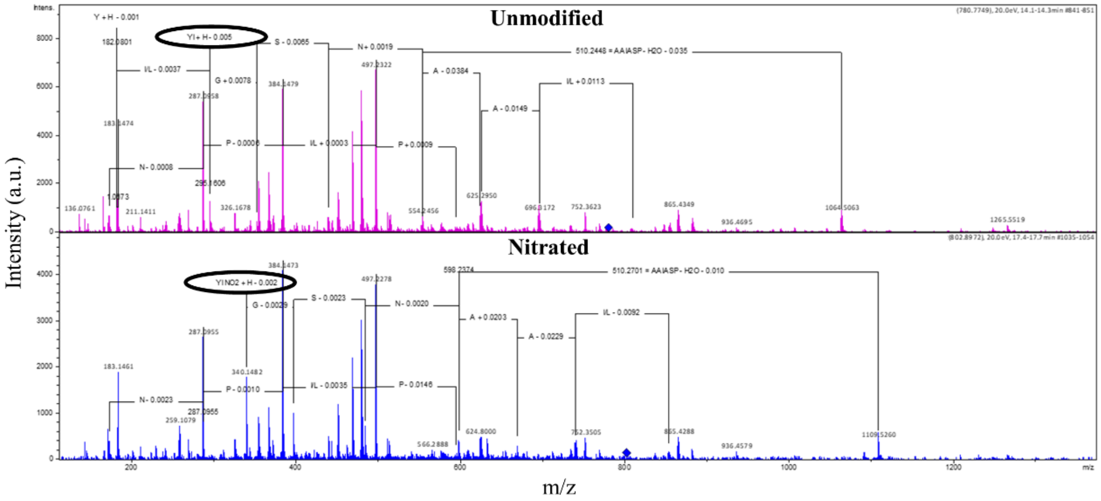

(B)

MS2-CP Nitration of Y-44 by ONOOH   Nitration mass is (NO_2) 45,992903- (H)1,007825 = 44.985078 Da

MS2-CP, peptide: 41- SQAYK –45

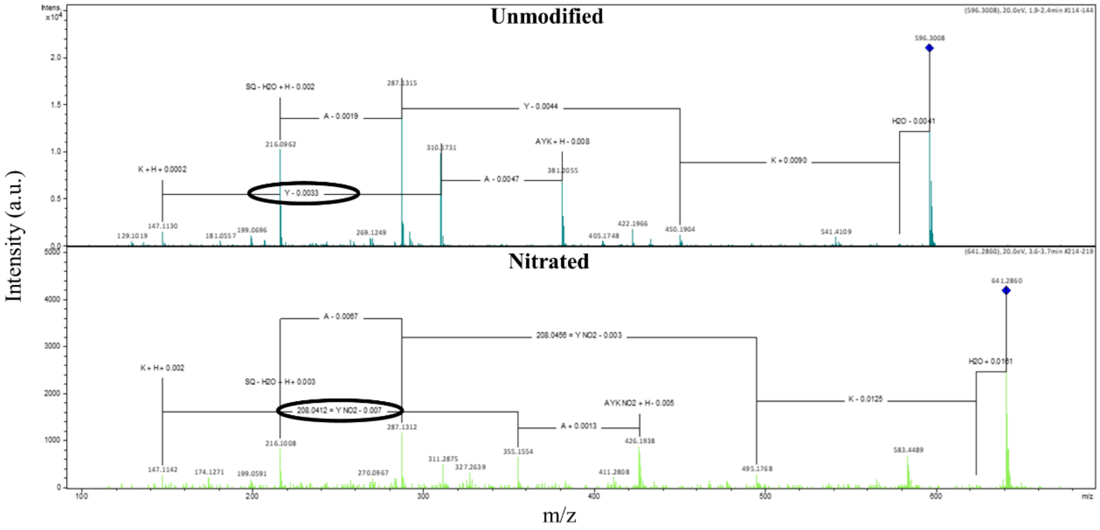

Supplemental figure 3: LC-ESI-Q-TOF and CID generated MSMS spectra of unmodified (upper panels) and nitrated (lower panels) MS2-CPs peptides containing in **A**, -Y130 and in **B**, -Y44. The sequence of each peptide is indicated above respective panels. Masses differences between peaks have been annotated with corresponding amino acids (in one letter code), beside the suggested identity of amino acids are indicated the difference of mass considered as compared to the theoretical masses of the indicated amino acid.

Supplemental Figure 4

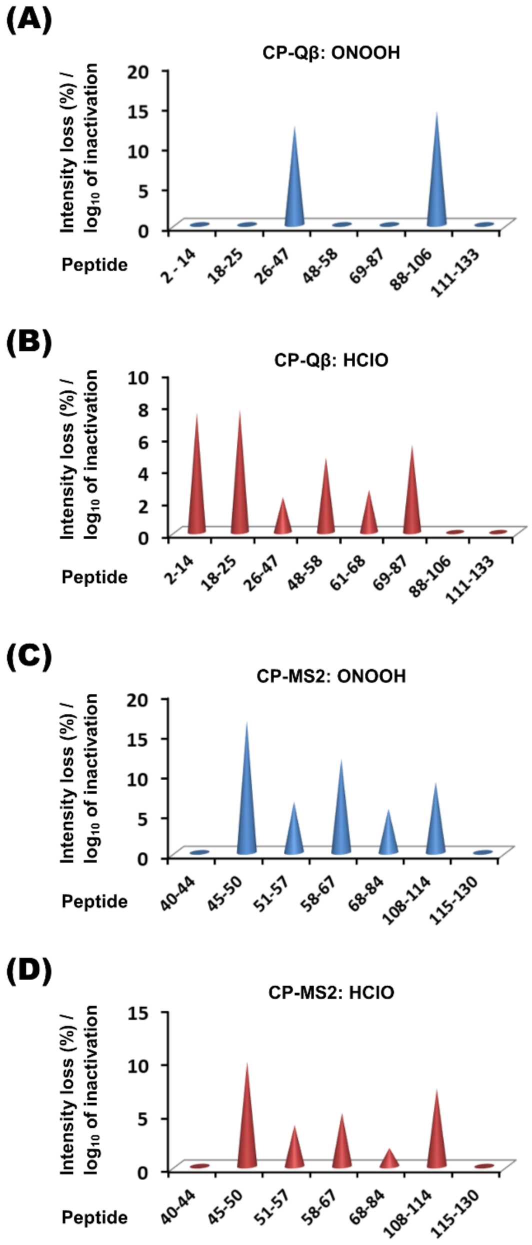

Supplemental Figure 4: Oxidants affect capsid proteins of Qβ and MS2 phages. Histograms showing the loss of intensities of CP regions of Qβ or MS2 per $\log_{10}$ of inactivation induced by HClO or ONOOH. **A**, Peptides of CP proteins of Qβ phages treated with ONOOH. **B**, Peptides of CP proteins of Qβ phages treated with HClO. **C**, Peptides of CP proteins of MS2 phages treated with ONOOH. **D**, Peptides of CP proteins of MS2 phages treated with HClO. In all panels, the position of each peptide is indicated and are reported peptides quantified three times out of three independent experiments using LC-ESI-Q-TOF. The intensities of the peptides were normalized to one CP peptide across all levels of inactivation per condition: peptides 69-87 and 111-133 for Qβ treated by ONOOH and HClO respectively, peptides 40-44 and 115-130 for MS2 treated with ONOOH and HClO respectively. The percentage of loss of intensities for each peptide were then compared to the condition under which phages were not inactivated by oxidants. The data are representative of three independent experiments.

Supplemental Figure 5

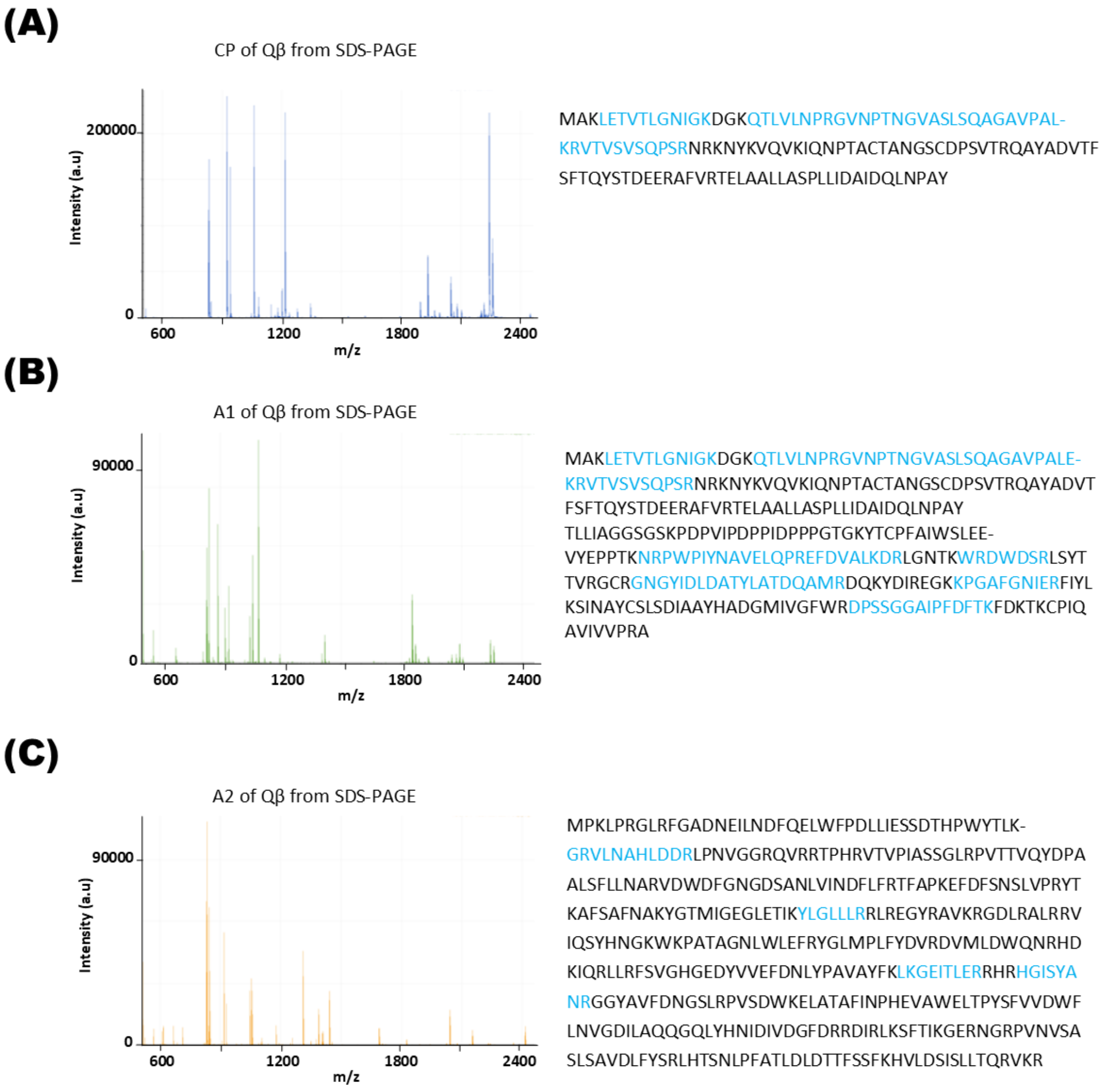

<u>Supplemental Figure 5</u>*: MALDI-TOF spectrum of Qβ proteins from SDS-PAGE.* **A**, The left panel displays the spectrum of Qβ phage CP protein excised from SDS-PAGE and digested by trypsin; the right panel displays the amino acid sequence of the protein. The letters in blue correspond to peptides recovered from the peptide mass fingerprint as obtained in the left panel. **B**, The left panel displays the spectrum of Qβ phage A1 protein excised from SDS-PAGE and digested by trypsin; the right panel displays the amino acid sequence of the protein. The letters in blue correspond to peptides recovered from the peptide mass fingerprint as obtained in the left panel. **C**, The left panel displays the spectrum of Qβ phage A2 protein excised from SDS-PAGE and digested by trypsin; the right panel displays the amino acid sequence of the protein. The letters in blue correspond to peptides recovered from the peptide mass fingerprint as obtained in the left panel. The data are representative of two independent experiments.

Supplemental Figure 6

(A)

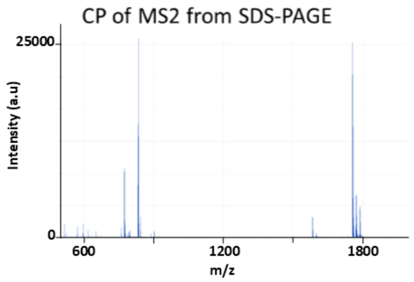

MASNFTQFVLVDNGGTGDVTVAPSNFANGVAEWISS-
NSRSQAYKVTCSVRQSSAQNRKYTIKVEVPKVATQTVGGVQLPVAAWRSYL
NMELTIPIFATNDDCALIVKAMQGLLKDGNPIPSAIAANSGIY

(B)

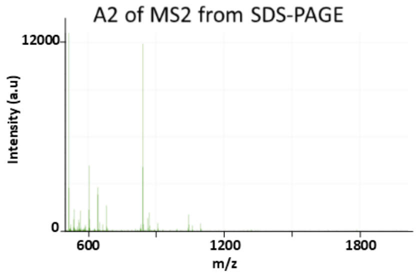

MRAFSTLDRENETFVPSVRVYADGETEDNSFSL-
KYRSNWTPGRFNSTGAKTKQWHYPSPYSRGALSVTSIDQGAYKRSGSSWG
RPYEEKAGFGFSLDARSCYSLFPVSQNLTYIEVPQNVANRASTEVLQKVTQG
NFNLGVALAEARSTASQLATQTIALVKAYTAARRGNWRQALRYLALNEDRK
FRSKHVAGRWLELQFGWLPLMSDIQGAYEMLTKVHLQEFLPMRAVRQVG
TNIKLNGRLSYPAANFQTTCNISRRIVIWFYINDARLAWLSSLGILNPLGIVWE
KVPFSFVVDWLLPVGNMLEGLTAPVGCSYMSGTVTDVITGESIISVDAPYG
WTVERQGTAKAQISAMHRGVQSVWPTTGAYVKSPFSMVHTLDALALIRQR
LSR

Supplemental Figure 6: MALDI-TOF spectrum of MS2 proteins from SDS-PAGE. **A**, The left panel displays the spectrum of MS2 phage CP protein excised from SDS-PAGE and digested by trypsin; the right panel displays the amino acid sequence of the protein. The letters in blue correspond to peptides recovered from the peptide mass fingerprint as obtained in the left panel. **B**, The left panel displays the spectrum of MS2 phage A2 protein excised from SDS-PAGE and digested by trypsin; the right panel displays the amino acid sequence of the protein. The letters in blue correspond to peptides recovered from the peptide mass fingerprint as obtained in the left panel. The data are representative of two independent experiments.

Supplemental Table 1

(A)

	Qβ protein identity			m/z signal intensities(a.u.) in the corresponding SDS-PAGE gel area (CP, CPx2, A1, A2 and HMs)														
	Protein	Peptide (aa number)		CP			CP x2			A1			A2			HMs		
m/z			HClO (µM)	0	670	1346	0	670	1346	0	670	1346	0	670	1346	0	670	1346
817.48	A2	264-270											100	200	50			
821.44	A1	199-205								2000	750	280					40	100
847.55	A2	160-166											10000	10000	2200			
917.47	A2	274-281											6000	9000	1500		50	140
940.55	CP A1	18-25		40000	40000	40000	4500	4500	18000	9000	12000	4000					300	1500
1020.46	A1	213-219								9000	17000	5000					60	100
1052.55	A2	43-53											250	450	70		15	30
1058.62	A2	262-270											500	600	100			
1059.58	CP A1	49-58		44000	44000	44000	4500	4500	18000	7000	5000	2000					150	1100
1088.58	A1	259-268								13000	13000	6000					300	1200
1092.56	A1	199-207								150								
1144.66	CP A1	4-14		3000	3000	3000	140	140	400	120	30	20					10	25
1215.68	CP A1	48-58		20000	20000	35000	450	1500	2500	1200	4500	2500					120	500
1438.68	A1	299-312								120	90	30						
1852.99	A1	184-198								5000	4000	1200					25	25
2080.1	CP A1	26-47		1200	1200	1200	20	20	90									10
2087.99	A1	230-248								70	20	10						

(B)

	MS2 protein identity			m/z signal intensities(a.u.) in the corresponding SDS-PAGE gel area (CP, CPx2, A2 and HMs)											
	Protein	Peptide (aa number)		CP			CP x2			A2			HMs		
m/z			HClO (µM)	0	670	1346		670	1346	0	670	1346	0	670	1346
524.32	CP	59-62		700	1700	2400		200	1800						
537.32	A2	187-190								800	700	100			
539.31	A2	191-195								1300	700				
571.35	CP	63-67		1500	2500	4000		250	4500						
596.32	CP	40-44		2000	2000	3500		100	4200						
652.41	CP	58-62		1000	1200	2200		100	2200						
664.35	CP	45-50		250	60	50									
724.37	A2	44-50								35					
760.44	CP	108-114		1500	1500	2500			350						
790.39	CP	51-57		500	400	800			1200						9
809.42	A2	3-9								120	25				
993.51	A2	178-185								160	40	60		5	40
1040.52	A2	90-99								220	40	500		10	300
1076.64	CP	59-67		60	170	300			200						
1177.6	A2	10-19										150			70
1320.61	A2	53-62								60					
1559.78	CP	115-130		100	60	140			200						
1565.84	A2	260-271										50		5	120
1595.89	A2	63-77		20	45	50			120						
1659.88	A2	132-147								20		150		9	250
1753.96	CP	68-84		45000	20000	25000		4000	35000					700	6000

Supplemental Table 1: *HClO and ONOOH induce the formation of CPx2 and HMs which are cross-links of Qβ and MS2 capsid proteins; the case of HClO*. **A**, Tables of Qβ peptides obtained from indicated bands excised (light blue) from SDS-PAGE and digested by trypsin, and analyzed by MALDI-TOF as a function of HClO concentration. **B**, Tables of MS2 peptides obtained from indicated bands excised (light blue) from SDS-PAGE and digested by trypsin, and analyzed by MALDI-TOF as a function of HClO concentration. The data are representative of two independent experiments. The numbers indicated are intensities (arbitrary unit). A blank box means not detected. The right column provides the identity of a peptide when predicted by theoretical trypsin digestion of the proteins.

Supplemental Figure 7

(A)

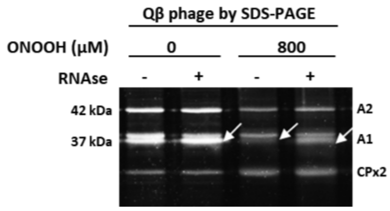

(B)

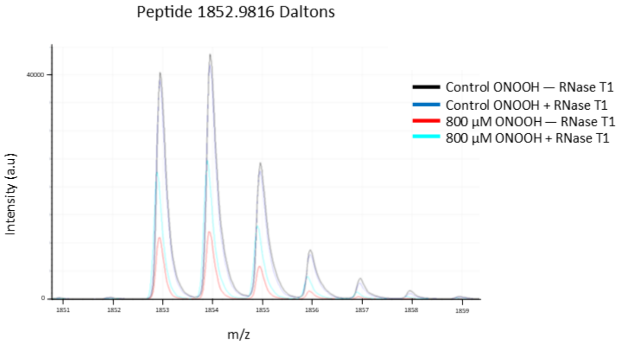

(C)

			Trypsin digest of A1 of Qβ (from SDS-PAGE)			
			Peptide Intensity (a.u.)			
			Control		ONOOH 800uM	
Sequence	Residues	mass	+DTT -RNAse	+DTT +Rnase	+DTT -RNAse	+DTT +Rnase
YDIR	252-255	566.2933	100	100	15	25
DWDSR	215-219	678.2842	100	79	11	11
FIYLK	269-273	683.4126	100	94	8	12
EFDVALK	199-205	821.4403	100	100	7	18
QTLVLNPR	18-25	940.5574	100	88	25	25
VTVSVSQPSR	49-58	1059.5793	100	82	27	42
KPGAFGNIER	259-268	1088.5847	100	108	42	58
LETVTLGNIGK	4-14	1144.6572	100	109	13	33
DPSSGGAIPFDFTK	299-312	1438.6848	100	100	8	13
NRPWPIYNAVELQPR	184-198	1852.9816	100	98	34	57
GNGYIDLDATYLATDQAMR	230-248	2087.9702	100	100	11	23
QAYADVTFSFTQYSTDEER	88-106	2257.9884	100	120	9	18

<u>Supplemental Figure 7</u>*: ONOOH cross-links A1 proteins to RNA genomes.* **A**, SDS-PAGE revealed by Oriole. Qβ phages were treated as indicated. Interestingly, the signal of A1 proteins strongly decreases with 800 µM of ONOOH and its intensity is partially rescued by the RNase T1 treatment. The intensity of A2 protein also decreases with 800 µM of ONOOH but it is not recovered by RNase T1. The data are representative of three independent experiments. **B**, Spectrum of the isotopic massif of the 184-198 peptides (1852.9816 Dalton) of A1 protein after excision from SDS-PAGE, digested by trypsin and analyzed by MALDI-TOF MS. **C**, Table showing A1 protein peptides recovered by SDS-PAGE coupled with MALDI-TOF MS; peptide intensities are shown as a function of the oxidant and RNase treatments of Qβ phage before being loaded on SDS-PAGE. The relative intensities were normalized into percentage of the intensities observed when the phages were not inactivated by ONOOH and treated by RNase T1.

Supplemental Figure 8

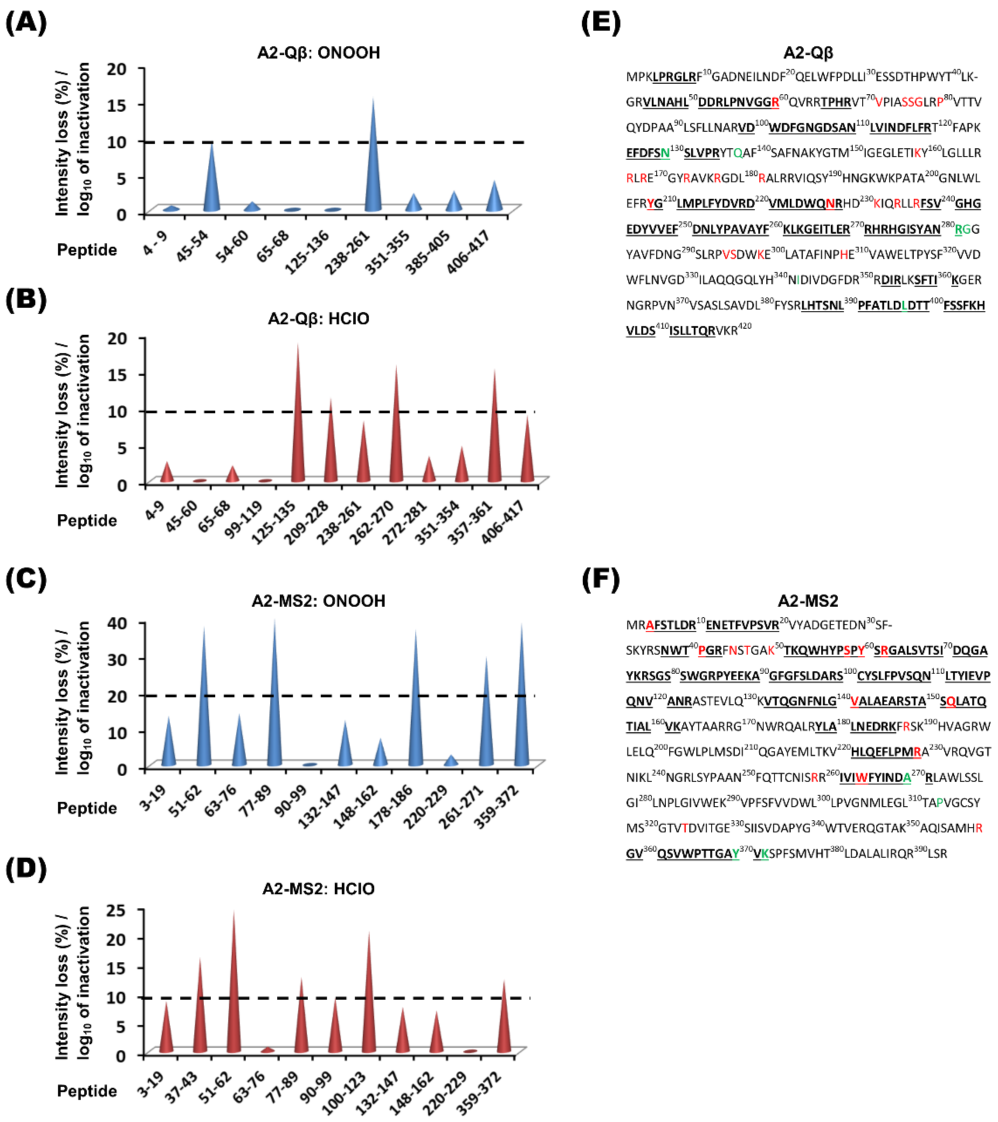

<u>Supplemental Figure 8</u>: *Oxidants affect preferentially peptides containing CYW at interaction sites*. Histograms showing the loss of intensities of A2 regions of Qβ or MS2 per log_{10} of inactivation induced by HClO or ONOOH. **A**, Peptides of A2 proteins of Qβ phages treated with ONOOH. **B**, Peptides of A2 proteins of Qβ phages treated with HClO. **C**, Peptides of A2 proteins of MS2 phages treated with ONOOH. **D**, Peptides of A2 proteins of MS2 phages treated with HClO. In panels A, B, C and D, the position of each peptide is indicated and are reported peptides quantified three times out of three independent experiments using LC-ESI-Q-TOF. The intensities of the peptides were normalized to one A2 peptide across all levels of inactivation per condition: peptides 65-68 and 45-60 for Qβ treated by ONOOH and HClO respectively, peptides 90-99 and 220-229 for MS2 treated with ONOOH and HClO respectively. The percentage of loss of intensities for each peptide were then compared to the condition under which phages were not inactivated by oxidants. The dash line arbitrarily separates preferentially affected to less affected peptides. The data are representative of three independent experiments. **E**, sequence of A2-Qβ. **F**, sequence of A2-MS2. In panels E and F, the amino acid numbers are indicated in superscript. The residues recovered by mass spectrometry reported in panels A, B, C and D are in bold and underlined. The residues which interact with CP or RNA genome are in green and red, respectively.

Supplemental Figure 9

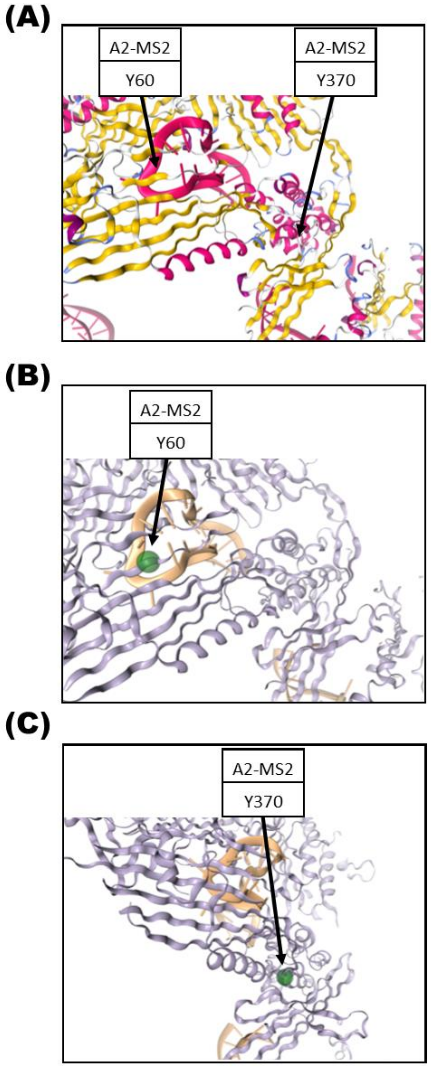

Supplemental Figure 9: *Peptides of the posterior side of MS2 phage A2 protein interact with CPs and the RNA genome.* **A**, Cartoon representation of the A2 protein sided by CP dimers and an RNA hairpin as viewed from above the protein and outside the capsid. Black arrows point at A2 residues which interact with CP proteins. α-helices are in pink, β-sheets are in yellow, loops are in blue, and undefined secondary structures are in white; the RNA hairpin is in hot pink. **B**, Cartoon representation of A2 protein in contact with the 3' terminal hairpin of the RNA genome of MS2. Y60 interacts with the 3' terminal hairpin of MS2. Y60 is represented by a green sphere, A2 and CPs are in violet, and the RNA is in beige. The black arrow points at Y60 of the A2 protein of MS2. **C**, Cartoon representation of A2 protein in contact with a neighboring CP. Y370 interacts with the neighboring CP. Y370 is represented by a green sphere, A2 and CPs are in violet, and the RNA is in beige. The black arrows point at Y370 of the A2 protein of MS2. All panels were made from PDB: 5TC1.

Supplemental Figure 10

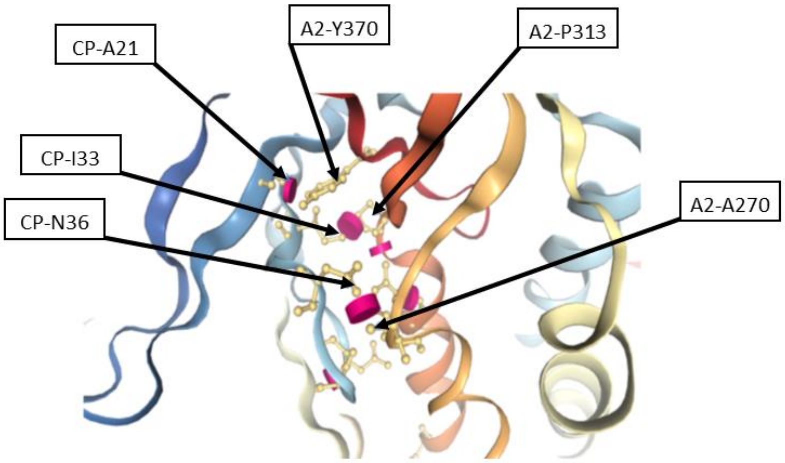

<u>Supplemental Figure 10</u>: The posterior side of the A2 protein of MS2 interacts with a CP. Cartoon and rainbow representation of A2 and CP proteins. The identities of the residues which are in close interaction are as indicated, black arrows pointing at their side chains which are in licorice representation. Hot pink disks are located at clashes between atoms. The panel was made from PDB: 5TC1.

Supplemental Figure 11

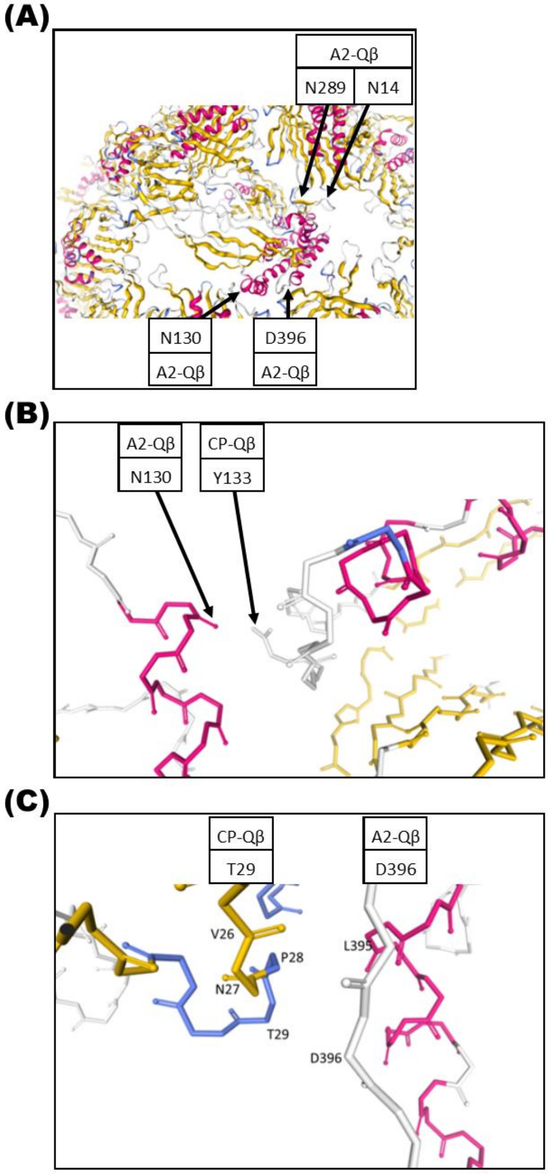

<u>Supplemental Figure 11</u>: *Peptides of the posterior side of Qβ phage A2 protein interact with CPs.* **A**, Cartoon representation of the A2 protein surrounded by CP dimers as viewed from above the protein and outside the capsid. Black arrows point at A2 residues which interact with CP proteins. **B**, Licorice representation of the 125-135 peptide of the A2 protein of Qβ phage which interacts with Y133 of the neighboring CP. Black arrows point at the two interacting residues N130 and Y133 of Qβ and CP, respectively. **C**, Licorice representation of the 385-405 peptide of the A2 protein of Qβ phage which interacts with T29 of a neighboring CP. For all panels, α-helices are in pink, β-sheets are in yellow, loops are in blue, and undefined secondary structures are in white. All panels were made from PDB: 5VLZ.

Supplemental Table 2

<u>Supplemental Table 2</u>: *HClO and ONOOH preferentially affect peptides interacting with sensitive amino acids C, Y and W. Cases of proteins Qβ-A2 and MS2-A2.*

		A2 - Qβ HClO		A2 - Qβ ONOOH		A2 - MS2 HClO		A2 - MS2 ONOOH		Total		Proportion	
		Damaged	Non damaged	Damaged	Non damaged	Damaged	Non damaged	Damaged	Non damaged	Damaged	Non damaged	Damaged (%)	Non damaged (%)
Interact, missing CYW	number (n)	**0**	**2**	**0**	**2**	**1**	**1**	**0**	**1**	**1**	**6**	**14**	**86**
	Peptide residues		**(45-60) (272-281)**		**(54-60) (385-405)**	**(37-43)**	**(3-19)**		**(3-19)**				
Interact with CYW	number (n)	**2**	**0**	**0**	**1**	**2**	**0**	**3**	**0**	**7**	**1**	**88**	**13**
	Peptide residues	**(125-135) (209-228)**			**(125-135)**	**(51-62)(359-372)**		**(51-62)(261-271) (359-372)**					
Do not interact missing CYW	number (n)	**2**	**4**	**1**	**4**	**1**	**3**	**0**	**4**	**4**	**15**	**21**	**79**
	Peptide residues	**(262-270) (357-361)**	**(4-9) (65-68) (351-354) (406-417)**	**(45-54)**	**(4-9)(65-68) (351-354)(406-417)**	**(90-99)**	**(132-147)(148-162) (220-229)**		**(90-99)(132-147) (148-162)(220-229)**				
Do not interact having CYW	number (n)	**0**	**2**	**1**	**0**	**2**	**1**	**2**	**1**	**5**	**4**	**56**	**44**
	Peptide residues		**(99-119) (238-261)**	**(238-261)**		**(77-89) (100-123)**	**(63-76)**	**(77-89) (178-186)**	**(63-76)**				

Supplemental Figure 12

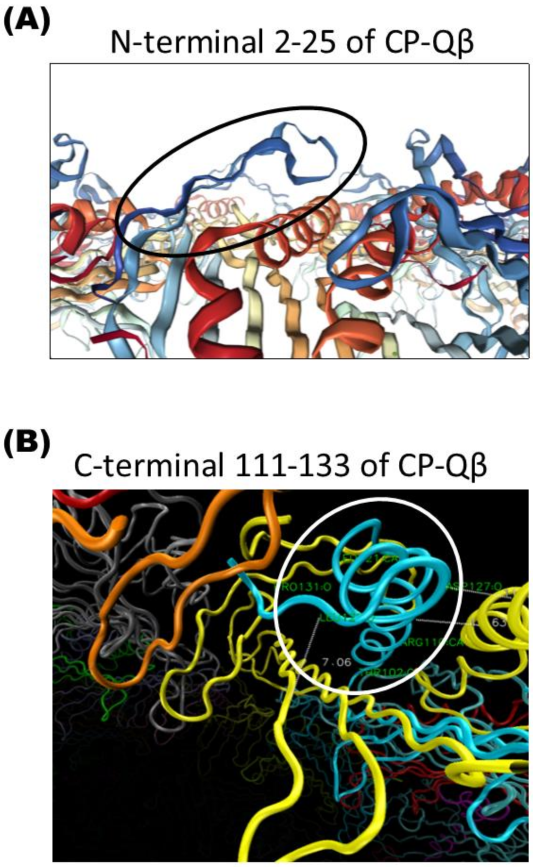

<u>Supplemental Figure 12</u>*: The 2-25 and 111-133 peptides of Qβ-CP do not interact with neighboring molecules*. **A**, Cartoon and rainbow representation of Qβ capsid. The 2-25 peptide of the CPs of Qβ forms a loop outside the capsid. The loop is highlighted by a black oval. **B**, Cartoon representation of the capsid of Qβ phage, each monomer of CP having its specific color. The 111-133 peptide of one monomer forms an α-helix on the outer side of the capsid, which is highlighted by a white oval; the distance to neighboring molecules is no less than 7 Å. The panels were made from PDB: 5VLZ.
